# RACIAL DISPARITIES IN COMMUNITY- AND INSTITUTION-BASED LONG-TERM SERVICES AND SUPPORTS

**DOI:** 10.1093/geroni/igz038.2867

**Published:** 2019-11-08

**Authors:** Chanee D Fabius

**Affiliations:** Johns Hopkins University, Baltimore, Maryland, United States

## Abstract

Long-term services and supports (LTSS) are services provided to individuals with functional limitations and chronic conditions who need assistance to perform daily activities such as bathing, dressing, preparing meals, and administering medications, and can be provided in community settings via services such as home health, as well as institutions such as nursing homes. Racial disparities are persistent across systems of LTSS, with older adults of color receiving lower quality care and experiencing worse health outcomes than their white counterparts. Given the increasing diversity of the aging population, and the need to ensure equity in quality and health outcomes in LTSS, there is a greater need for more understanding of how experiences of care vary across multiple settings for diverse groups of older adults and the people who help them. This symposium will feature 5 presentations that provide novel insight regarding racial disparities in community- and institution-based LTSS. We focus on racial differences in functional needs and disparities among those receiving home health services and living in nursing homes. Individual presentations will describe 1) race and gender differences in physical functioning needs of older adults; 2) disparities in home health quality across racially diverse and low income geographic areas; 3) racial disparities in nursing home residents overtime; 4) racial and ethnic disparities in rates of 30-day rehospitalization from skilled nursing facilities among Medicare Fee-For-Service and Medicare Advantage patients; and 5) the impact of the unequal burden of care provided to minority nursing home residents by staff of color.

